# Reviews and Book Notices

**Published:** 1878-04

**Authors:** 


### Reviews and Book Notices.

PRACTICAL GYNÆCOLOGY: A Handbook of the diseases of Women. By Heywood Smith, M. A., M. D., Oxon. With Illustrations. Philadelphia: Lindsay & Blakiston. 1878.

The author of this volume states in the preface that his "object in the present work has been to present the busy practitioner with a book systematically arranged, burdened with no discussions on vexed questions of pathology, and giving at a glance the salient points of diagnosis and treatment with clearness and brevity." In all these respects he has fully succeeded; and, although the book is a small octavo of only 205 pages, he has managed, after devoting 21 pages to a description of the means and methods of making abdominal and pelvic examinations, 15 to a Table of Contents and 14 to an Appendix of Remedies andan Index, to consider the causes, symptoms, signs, diagnosis, prognosis and treatment of 108 different diseases. Of course, such a result could only be accomplished by a vigorous application of the "boiling down" process. There is probably not a redundant word in the book. As examples of the author's condensing ability, it may be stated that he disposes of the subject of Menorrhagia in less than a single page, and of Dysmenorrhea in all its varieties, in one page and a half!

The systematic arrangement of the book is admirable, and might be profitably followed by some of the larger and more pretentious works on diseases of women. Chapter I. is given to the Means of Diagnosis; chapter II., to General Diseases, or those of constitutional character, including Syphilis, Cancer and Phthisis; chapter III., to Diseases of the Ovary; chapter IV., Diseases of the Oviduct; chapter V., Diseases of the Broad

Ligaments; chapter VI., Diseases of the Unimpregnated Uterus; chapter VII., Diseases of the Vagina; chapter VIII., Diseases of the Vulva; chapter IX., Diseases of the Mamma; chapter X., Functional Diseases; chapter XI., Diseases Connected with Pregnancy; chapter XII., Diseases Connected with Parturition; chapter XIII., Diseases Consequent on Parturition.

Our author deserves our thanks for giving us a much-needed word, "Spammenorrhea," to express scantiness of the menstrual flow, as distinguished from its absence, both these conditions having been heretofore included in the term Amenorrhea, a word clearly applicable only to those cases in which there is no menstrual secretion.

On the mooted question, Dilatation *vs.* Incision, in the treatment of stricture of the cervical canal, at whatever point the constriction may be, Dr. Smith is in favor of the knife; and states his preference for the single-bladed hysterotome. He advises that the incisions should not be deep, and that they should be followed immediately by forcible dilatation.

It is a significant sign of the tendency of surgical opinion in regard to large uterine fibroids, that our author, speaking of the subperitoneal variety gives, under the head of Treatment, only one sentence, "where so large as to threaten health, removal by abdominal section." Likewise, he says, in reference to intra-uterine fibroids which for any reason cannot be successfully dealt with by other means, and where the presence of either pain, hemorrhage or rapid growth renders the patient's life unendurable or prevents her from pursuing her necessary avocations, that the total extirpation of the uterus and its morbid contents is advisable; providing only, that a more or less healthy cervix can be left as a stump, as the attempted removal of the cervix also would be attended with too great risk, owing to its relation with the bladder. And he draws attention to the fact that, although the operation of hysterectomy (he prefers this word to "hysterotomy" and "gastrotomy," as being more accurate, just as, for the same reason, he advises "ovariectomy" instead of "ovariotomy") is perhaps the most formidable in uterine surgery, the mortality after removal of the uterus is not greater than obtained in the early history of ovariotomy.

Two conditions are pointed out in which the author regards the process of "produced sloughing" as applicable to the treatment of intra-uterine fibroids; "one where the tumor is sessile and presents considerable difficulties to the process of enucleation, and the other where the uterus is so enlarged and misplaced by a tumor which may be mainly interstitial, as that the os uteri is tilted upward and jammed against or above the os pubis. In the former case, it is suggested to bore a deep hole into the most depending portion of the tumor through the os uteri by either the actual cautery or potassa fusa, opening thereby the capsule of the tumor. In the latter, it is recommended to, in fact, bore a new os uteri, as it were, by the actual cautery in the most dependent portion (the posterior) of the uterus itself. It has been successfully carried out; the tumor sloughing and disintegrating has been expelled gradually through the artificial os, and as the tumor thus becomes lessened, the uterus assumes its natural position, the os comes down from its former situation, and the hole through which the tumor has sloughed out eventually heals up."

For the reversion of the chronically inverted uterus, Dr. Smith gives decided preference to the plan of Dr. Noeggerath, of New York, which consists in feeling for the insertion of one of the oviducts and making steady pressure upon the spot with a finger, counter-pressure being at the same time made on the outside, or occasionally by pressing the uterus against the promontory of the sacrum. He regards this as by far the most scientific method of procedure, because the uterine walls are thinner in this situation than at any other point, and because, also "as the insertion of the oviduct is situated laterally, any gain of reversion is first made in that portion that intervenes between the insertion of the oviduct and the cervix; and the reinvagination of the cervix once having been begun, steady pressure will soon carry the whole of the organ upward in an oblique direction."

On the whole, the book is a good one, but who wants it? Even the busy practitioner, for whose needs it has been especially written, when desirous of referring to a particular subject, always prefers a full reference to a scanty one; so that if he had the work before us, he would still need the larger work of Thomas, or Hewitt or Barnes; and if he were in possession of either of these he would surely have no need for this "Handbook."

We cannot help thinking that in his desire to do justice to his father, Dr. Protherae Smith, and to show his filial regard, our author has somewhat overstepped the bounds of propriety. The book is dedicated to his father, and with this we find no fault; but, in addition to this, there are in the body of the work no less than ten allusions to "my father" and his superior instruments, apparatus and plans of procedure. Of course, this may be considered only a matter of taste, but it seems to us to be not one of good taste.

A. R. J.

Diseases of the Nasal cavity and the vault of the Pharynx. Translated from the German of Dr. Carl Michel, of Cologne, on the Rhine. First American edition. Detroit, Michigan: C. Jung, 1877; pp. 109.

This pamphlet is not a complete treatise on the diseases of the nose, but an exposition of the author's views and methods of treatment as they have been formulated during a long practice. In the first place, he emphatically insists upon a thorough examation and justly denounces the slip-shod manner in which nasal affections generally are disposed of by physicians. The nasal cavity permit of an inspection of most of its parts as well as other cavities, and their diseases can successfully be treated only by local treatment under the guide of proper specula and good light. The treatment of chronic nasal catarrh by galvano-cautery is considered by Michel superior to any other. He believes ozæna is a chronic purulent inflammation of the sphenoidal and ethmoidal cells, and fortifies his view by very plausible reasons.

This brochure was published in 1876; it was printed on inferior paper and presented in a very unfinished dress like all the continental pamphlets and periodicals. In this respect the American edition is far superior to its original. And this is all we have to say in its favor. It is a very unpleasant duty to condemn; but in justice to the author we are compelled to say that the translation is a very unskillful and awkward reproduction of the original. The translator is utterly incapable of doing the work well, for two reasons, viz., he cannot construct a grammatically correct English sentence, and has no knowledge of medical matters. Every paragraph in the translation is an offense against English grammar; every page contains violations of orthography; every chapter is rendered unintelligible by the wrong application of words which convey a meaning different from that which Dr. Michel expressed in his original article.

The justice of this criticism may be estimated by a consideration of the following instances, selected from a large number of sentences: Page 19, "If the stage of muco-purulent secretion does not stop otherwise, then insufflations of nitrate of silver one part to twenty of talc., which is done by means of a strong glass pipette, one end of which is cut out shovel-like, so that the powder may be easily taken up—while the other end is curved so that the insufflation can be accomplished from through the mouth." We confess we had to consult the German text to understand this.

On page 15, we read *glandular bed* for *Drüsenlager* (a layer of glands); page 17 *tumor* for *Geschwür* (ulcer); page 30, *bluestone* for *Höllenstein* (solid silver nitrate); page 41, *cavities of the septum* for *Siebbeinhöhlen*, (ethmoidal cells); page 46, *spray* for *Spritze* (syringe); page 35, an *odorous* nose for *Stinknase* (fetid nose)! And on page 80 where Dr. Michel wishes to say that mercurial ointment (unguentum cinereum) should be used, the translator speaks of "embrocations of ciner salve (*ungt. ciner*—ash-ointment)"!

We are exceedingly sorry that we find so little to praise and so much to censure. But to make the translation intelligible it must be revised and re-written. F. C. H.

Handbook of the Practice of Medicine. By M. Charteris, M. D., Professor of Practice of Medicine, Anderson's College, Glasgow; and Physician and Lecturer in Clinical Medicine, Glasgow Royal Infirmary. Philadelphia: Lindsay & Blakiston, 1878; pp., 225. Chicago: Jansen, McClurg & Co. Price $2.

This compendium is chiefly devoted to the symptoms, pathology and treatment of those diseases that most frequently engage the attention of the practitioner. The work reveals its author as a physician of large attainments and experience, and possessing the rare capacity of briefly stating what is most essential to be known, relating to the different subjects of which he treats. The book will be valuable to students—for whose use it is especially designed—as it clearly outlines to them interesting subjects of investigation; and to physicians also, by refreshing their memories on points to which they may not for some time have given especial study.

The book contains a number of pages of formulæ; and a number of illustrations—one of especial excellence as explaining cardiac murmurs and the valvular action of the heart.

